# Adolescent depression: Study protocol for a randomized, controlled, double-blind multicenter parallel group trial of Bright Light Therapy in a naturalistic inpatient setting (DeLight)

**DOI:** 10.1186/s13063-018-2949-0

**Published:** 2018-10-19

**Authors:** Martin Holtmann, Laura Mokros, Inken Kirschbaum-Lesch, Michael Kölch, Paul L. Plener, Christian Ruckes, Michael Schulte-Markwort, Tanja Legenbauer

**Affiliations:** 1 Clinic for Child and Adolescent Psychiatry, LWL University Hospital Hamm of the Ruhr-University Bochum, Heithofer Allee 64, 59071 Hamm, Germany; 2 Department Child and Adolescent Psychiatry and Psychotherapy, Brandenburg Medical School, Neuruppin, Germany; 3 Department of Child and Adolescent Psychiatry and Psychotherapy, Ulm University, Ulm, Germany; 4 Interdisciplinary Centre for Clinical Trials, Mainz University, Mainz, Germany; 5 Department Child and Adolescent Psychiatry, Hamburg University, Hamburg, Germany

**Keywords:** Depression in youth, Treatment, Bright light therapy, Inpatients

## Abstract

**Background:**

Depressive disorders are among the most prominent health problems in youth. Even with the best available pharmacological and non-pharmacological treatments, remission rates are low. Without early treatment, depression in youth is associated with a high risk of symptom progression, chronicity, co-morbidity, and suicidal behavior. Thus, adolescent depression is a prime candidate for innovation in treatment. In depressive adults, meta-analytic evidence has proven that bright light therapy (BLT) is a potent low-threshold intervention, promising due to easy application, low side effects, and optimized compliance. In adolescents, studies with small samples show promising effects. This randomized controlled trial will examine the effectiveness of BLT in youth inpatients.

**Methods/design:**

In this randomized, controlled, double-blind multicenter parallel group trial, morning BLT is applied for four weeks in addition to treatment as usual (TAU) for depressed youth inpatients (daily morning exposure to bright light via light-emitting glasses, 10,000 lx, for 30 min) and will be compared to a control condition (placebo light treatment, red light, identical light glasses). The primary objective is to assess whether BLT reduces symptoms of depression in youth with greater effect compared to placebo light therapy. Secondary objectives are to examine the impact of BLT on responder status, application of antidepressant medication, and further depression-related symptoms (sleep, activity, quality of life, satisfaction with health, general psychopathology, alertness, and circadian function). *N* = 224 patients will be recruited in a naturalistic inpatient setting. A follow-up will be carried out after three and six months.

**Discussion:**

The study aims to discuss and evaluate BLT as an additive method supporting standardized clinical procedures dealing with severe to moderate depressive symptoms in youth.

**Trial registration:**

German Clinical Trials Register, DRKS00013188. Registered on November 30, 2017.

**Electronic supplementary material:**

The online version of this article (10.1186/s13063-018-2949-0) contains supplementary material, which is available to authorized users.

## Background

Depressive disorders are among the most prominent health problems in youth. In adolescents, prevalence rates of depression are in the range of 5–6% [1], with a lifetime prevalence rate of 20% by late adolescence [2]. Prevalence in girls is twice as high as in boys and higher symptom load is found in youth with a high number of psychosocial risks [3]. The five-year recurrence rate in adolescent depression is as high as 40% [4]. In addition, a depressive mood during the winter season is quite common among youth in central Europe, with at least 2% of adolescents and young adults meeting criteria for seasonal affective disorder (SAD), e.g. a clinically meaningful depression with a seasonal pattern [5]. Of all depressive youth, 10% receive inpatient treatment at least once. In Germany, inpatient treatment rates for adolescent depression have increased dramatically by > 200% from 2000 to 2007, compared to 38% for all mental disorders [6].

Adolescent depression may interrupt educational attainment and thus affect human capital accumulation and future earnings. Since teenage suicide, for which depression is a principal risk factor, is the second leading cause of death in German youth, preceded only by traffic accidents [7], it is important to acknowledge that the human costs associated with depression are in a real sense greater than the economic costs. Adolescent depression is a key risk factor for adult depression; it severely impairs work and school management, leisure activities, and social contacts; frequency and degree of disabilities in depressed youth are at least as pronounced as in adults [8]. In short, depression in adolescents is prevalent, of significant public health importance, and thus a prime candidate for innovation in treatment.

Despite a huge increase in treatment research in the past 20 years, remission rates of pharmacological treatments are low [9]. In the carefully designed gold standard studies TADS and TORDIA, remission rates for moderate to severe depression were < 40% after three and six months of combined medication and psychotherapy ([10, 11]). A recent meta-analysis yielded only small effect-sizes for selective serotonin reuptake inhibitor (SSRI) and serotonin and norepinephrine reuptake inhibitor (SNRI) treatment of depressive disorders in children and adolescents [12]. Treatment decisions are further complicated by safety concerns of antidepressant-related self-harm and suicide in youth [13]. Despite these problems, a doubling of prescriptions for antidepressants in German adolescents has been observed in recent years [14]. The current evidence- and consensus-based clinical practice guideline of the German Association for Child and Adolescent Psychiatry (DGKJP) for the treatment of adolescent depression concluded that due to “the glaring lack of clinical studies (…) there is a pressing need for intervention research” [15].

Within the last decade, especially in the field of adult psychiatry, evidence suggests promising effects of bright light therapy (BLT) with respect to the improvement of depression symptoms. We conducted a systematic overview regarding BLT for the treatment of both non-seasonal and seasonal depression by using a broad range of databases. The present search is an update of already published reviews by our group laying special emphasis on clinical trials of BLT in depressed adolescents ([16, 17]). In adults, recent meta-analytic evidence [18] has proven that BLT leads to a significant reduction of depressive symptoms in non-seasonal depression (SMD = − 0.62, *P* < 0.001). In particular, BLT appears to be efficacious when administered for 2–5 weeks (SMD = − 0.78, *P* < 0.001). The authors emphasize the obvious need to optimize the duration and intensity of exposure and the timing and duration of treatment sessions. In a Cochrane review on BLT in non-seasonal depression, studies with a higher methodological quality rating showed unequivocal superiority of BLT over control treatment (effect-size 0.90, 95% confidence interval [CI] 0.31–1.50 [19]). Of note, treatment duration in almost all included studies was brief (1–2 weeks). Existing evidence suggests that longer treatment (at least four weeks) yields larger effects.

Up until now, only very few studies investigated the effects of BLT on depression in youth (see [20] for a systematic review of studies until 2012 and [18] for meta-analysis including more recent trials). In one of our pilot studies [21], remission rates after two weeks of BLT (light box, 10,000 lx; *n* = 30) and a further three weeks of follow-up were 46.7% for BLT and 25.9% for dim light (100 lx; *n* = 27); however, these differences were not significant due to the small sample size. BLT significantly improved sleep difficulties in youth [21]. Sleep restoration occurred only during BLT and remained stable during follow-up, while dim light remained without any effect. BLT may, therefore, be especially suited for depressive adolescents, of whom 75% are affected by problems with the sleep-wake rhythm [22]. A combination of BLT and an additional night with sleep restriction did not result in greater symptom reduction than BLT alone in 60 adolescent depressive inpatients [23]. A secondary analysis with actigraphy and sleep diary data showed greater sleep-enhancing effects in the BLT group than the combined group after two weeks of intervention [24].

BLT is promising due to robust evidence in adults, easy application, low side effects, and heightened compliance [25]. Light glasses have been shown to lead to the same results as a 10,000 lx light box ([26, 27]). However, empirical evidence on BLT for youth is scarce and pilot studies suffer from small sample sizes, lack of randomization, shortness of treatment duration, and lack of control settings. Moreover, a combination of BLT and treatment as usual (TAU; including pharmacotherapy) might be promising, because more naturalistic studies taking into account antidepressants do not exist. The proposed trial shall extend the pilot findings; treatment duration will be prolonged to four weeks to yield larger effects and follow-up will be extended to six months. Results of the proposed trial should allow conclusions as to the acceptability, the effects, and the costs of BLT in youth, thus providing a basis for decisions as to the broader implementation. We assume that BLT should lead to more robust symptom remission, potentially facilitating dose reduction or even replacement of antidepressants and a subsequent lower risk of adverse events (AEs). BLT may lead to a faster onset of effect [28], leading to shortened hospitalization, and can prevent transition to more severe forms of depression.

The projected trial will:

- examine whether BLT (daily morning exposure, 10,000 lx, for 30 min) in addition to multimodal TAU reduces symptoms of depression in youth inpatients with greater effect compared to placebo light therapy plus TAU (red light);
- assess the impact of BLT on responder status, application and dosage of antidepressant medication, and further depression-related symptoms (sleep, activity, quality of life, satisfaction with health, general psychopathology, alertness, circadian function), and length of stay in hospital;
- assess the prevalence and severity of BLT side effects which have been vastly neglected in previous studies.

## Methods/Design

### Study rationale

This trial is a randomized, controlled, double-blind multicenter parallel group study. The trial protocol has been approved by the medical ethics committee of the Ruhr-University Bochum, Germany (registration number 17–6140-BR) and is registered with the German Clinical Trials Register (DRKS00013188). The trial will be conducted according to the principles of ICH-GCP and appropriate legal regulations. The Professional Code for Physicians in Germany and Declaration of Helsinki in its actual version will be adhered to. For recommended items to address in a clinical trial protocol according to the SPIRIT 2013 Checklist, see "Additional file 1". 

### Study setting and recruitment

Four Departments of Child & Adolescent Psychiatry of the Universities/Medical Schools of Bochum, Ulm, Hamburg, and Brandenburg/Neuruppin participate in the study and will recruit inpatients. The study sample should reflect everyday clinical practice to be as representative as possible for inpatient youth (aged 12–18 years) with moderate to severe depression [29]. Diagnoses will be ascertained by structured clinical interviews. To maximize generalizability (i.e. testing for effectiveness), exclusion criteria are limited to situations that would prevent full participation in the study or that might require additional treatment incompatible with study treatments. Inclusion and exclusion criteria are listed in Table 1. Antidepressant medication is allowed and controlled for in the analyses performed (naturalistic approach).

**Table 1:** Inclusion and exclusion criteria

Inclusion criteria	Moderate to severe depression (BDI-II)
	Inpatients aged 12–18 years
	Written informed consent of the inpatient and the caretaker
Exclusion criteria	Acute suicidality, bipolar 1 disorder, or schizophrenia
	Pregnancy or lactation
	Treatment with beta-blocker or with high-potency neuroleptics
	Diseases of the retina
	IQ < 70
	Non-German-speaking child or caretaker

*BDI-II* Beck Depression Inventory II

### Informed consent process

First, patients and parents will be informed in written and oral forms regarding the study, the procedures, and the potential risks or discomforts and potential benefits. An informed consent document with details about the study including its purpose, duration, procedures, and key contacts, as well as risks and potential benefits, will be handed out to patients and parents. The patients and parents will then decide whether to give written consent. Patients or parents unwilling or unable to consent will not be included in the study. At any time, participants or parents may withdraw their consent for any reason without any negative consequences regarding their treatment.

### Randomization and blinding procedures

Potential bias is minimized by randomized treatment allocation and by blinded outcome assessment. Since patients will be blinded towards their treatment, patient-reported outcomes including the primary outcome will be blinded. Independent raters unaware of the patient’s treatment will perform further assessments (CDRS-R). Randomization is performed using the electronic case report form and will be stratified by prior pharmacological antidepressant therapy (yes/no), trial site, and sex. The randomization ratio will be 1:1 based on blocks of variable length.

Patients and clinical raters will both be blinded regarding treatment allocation. While the primary outcome (BDI-II) will be rated by the blinded patients themselves, the clinical evaluation (CGI-I, CDRS-R) will be performed by the blinded clinician. Blinding will be assured by separating diagnostic procedures from general patient and data handling. There will be one research assistant organizing recruitment and all patient-relevant data handling as well as a second person solely performing the diagnostic assessments. This procedure has proven to be feasible in our pilot study [21].

### Experimental / control condition

Finding an appropriate control condition for light therapy to minimize placebo effects is difficult, since these effects are expected to be quite high in depression. In the projected study, the active light treatment will consist of daily morning exposure to white fluorescent light (light glasses Luminette®; 10,000 lx) for 30 min for 28 days (weekends excluded), while the placebo treatment will be provided by identical glasses with a red light without known effects on depression. This design has been shown to be feasible in adolescents [21]. Since light therapy is most effective if applied at an optimal time in the circadian phase of the patient, light interventions will be carried out 7.5–9.5 h after the individual evening melatonin onset (DLMO) estimated by the Morningness–Eveningness Questionnaire (MEQ) [30, 31].

### Interventions

Following feedback from the participants in our pilot studies, light boxes are replaced by light glasses that allow patients to carry on with everyday tasks and fit into their daily routine while giving the same results as a 10,000 lx light box ([26, 27]). To ensure an appropriate and constant number of lux administered in each condition, intensities are quantified using a Lux meter (PCE-172). If a patient is not able to receive the intervention on a given day (e.g. due to illness), he is committed to prolong the intervention phase for one day. In case of several missing interventions (max. 20% of the sessions), the patient will be excluded from the study. In order to control for placebo effects, the therapy expectancy and credibility questionnaire will be administered in a modified version before treatment.

Study interventions will be added to a naturalistic multimodal inpatient TAU. TAU will be documented in detail: due to the requirements of the recently introduced new reimbursement system for psychiatric hospitals in Germany, the participating centers are already documenting continuously type, frequency, and duration of patient-directed interventions electronically for all relevant professions (psychiatrists, psychotherapists, nurses, occupational therapists, etc.). These data can be aggregated for participants of the projected study to determine the distribution among groups.

In some cases, psychopharmacological antidepressant treatment may be warranted and will be provided according to the attending physician’s clinical judgment (non-interventional). Afternoon outdoor activities are part of a comprehensive youth inpatient care but are unlikely to interfere with the antidepressant response to BLT, since treatment response is maximized if BLT is initiated no later than 8.5 h after a patient’s estimated melatonin onset, e.g. only in the early morning. Light exposure at midday or in the afternoon does not result in an antidepressant effect [32].

### Intervention scheme / trial flow

Inpatients from all trial sites that meet the inclusion criteria and give informed consent are consecutively included into the trial within one week after admission. After inclusion, inpatients will be interviewed by the blinded research psychologist. Initial self-report measurements and objective data in relation to depression and associated co-morbidity as well as medication are assessed by a trained diagnostician. In addition, treatment expectancy and responsibility to seasonal effects will be assessed. After the pre-treatment assessment (T1), patients are randomized to either a placebo control condition (PCC) or BLT. Both groups also receive TAU. Trial interventions will be delivered each morning (except weekends) over a period of four weeks about 7.5 h after the DLMO assessed with the MEQ.

**Fig. 1:**
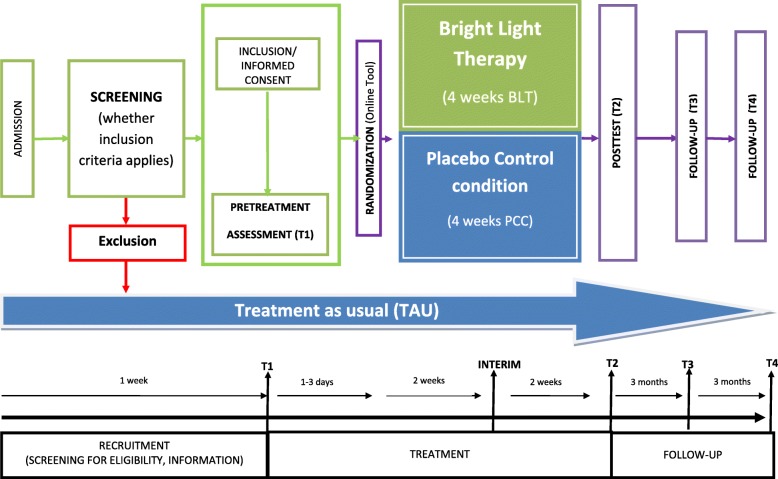
Trial flow overview. PCC placebo control condition + TAU, BLT Bright Light Therapy + TAU; T1 assessment before treatment, T2 assessment at the end of the four-week treatment, T3 follow-up after three months

**Fig. 2:**
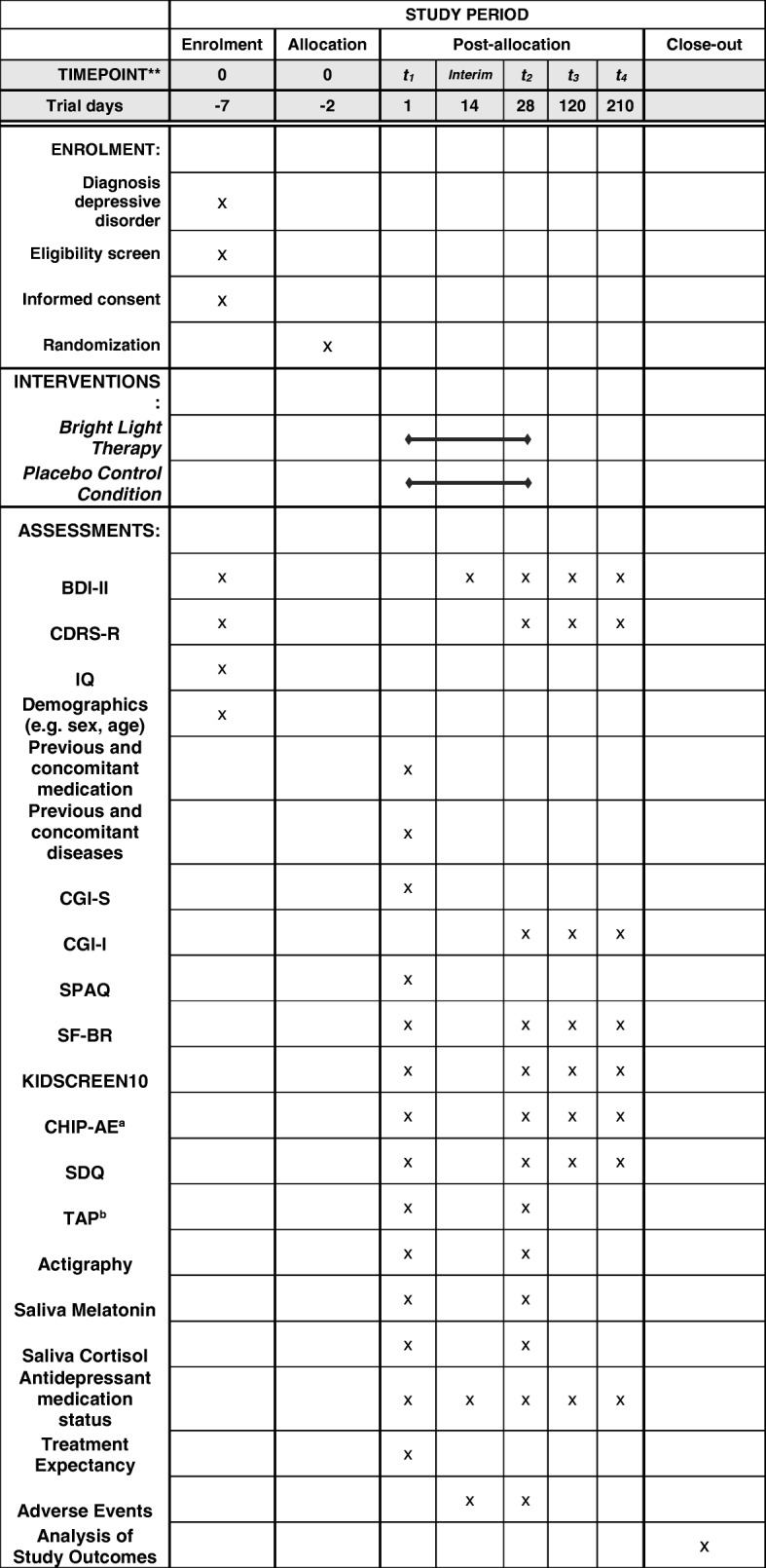
Study process schedule (according to SPIRIT guidelines). T1 pre-treatment assessment, T2 post-treatment assessment, T3 follow-up three months after T2, T4 follow-up six months after T2, BDI-II Beck Depression Inventory II, CDRS-R Children’s Depression Rating Scale-Revised, CGI-S Clinical Global Impression-Severity, CGI-I Clinical Global Impression-Improvement, SPAQ Seasonal Pattern Assessment Questionnaire, SF-BR Sleep Questionnaire-B/Revised, KIDSCREEN10 Health Related Quality of Life self-rating assessment – short version, CHIP-AE Child Health and Illness Profile-Adolescent Edition, SDQ Strength and Difficulties Questionnaire, TAP Testbattery for Attentional Performance; ^a^only subdomain physical activity administered; ^b^ only Alerteness subtest administered

**Fig. 3:**
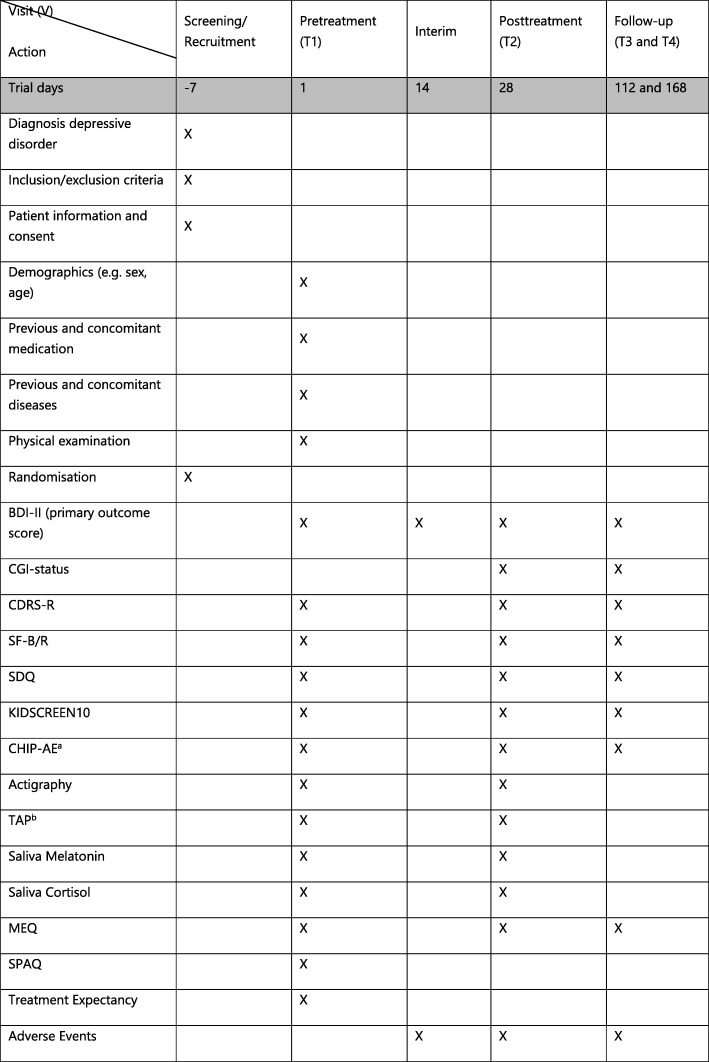
Overview of time-points of visits and procedures per time-point

During the intervention trial, an interim assessment with the BDI-II after two weeks will take place in order to control patient-perceived changes in depression more closely. There is no full assessment in order to reduce stress for the patients. Four weeks after completion of the treatment, individuals will again be interviewed by the blinded research psychologist and score the self-rating questionnaires as well as perform the neuropsychological assessment and deliver saliva melatonin and cortisol (post-treatment assessment; T2). Three and six months after T2, follow-up assessments will be performed via telephone and questionnaires to control for stability (T3 and T4). There is no full reassessment for neurobiological and neuropsychological data in order to facilitate the response to fulfil the follow-up assessment and to meet patients’ needs best. Moreover, AEs will be assessed in both treatment groups on a weekly basis. For an overview of the trial flow, see Fig. 1. For details on procedures per time point, see Figs. 2 and 3.

### Measurements

The primary endpoint of the study is the change in patient-perceived severity of depressive symptoms after treatment, assessed with the BDI-II.

#### Beck Depression Inventory-II

The BDI-II is a self-rating instrument that is extensively used in clinical studies worldwide to assess the degree of depressive symptoms of the last seven days (validated in German adolescents; [33]). The score is easily calculated as a sum score. This score will be used in a dimensional way in the analysis, as this provides more power than a categorical use. According to NICE guidelines, the clinically relevant difference between treatment groups is 3 BDI-II scale points. The BDI-II standard deviation is expected to be about 10 scale points. The study aims to show the same treatment differences as in our pilot study [21] of about 4.37 scale points, which is clearly a relevant treatment difference.

The secondary endpoints include independent evaluator-, adolescent-, parent-, and clinician-rated measures of depression, improvement and impairment, and functional status. In addition, measures are included to allow the exploratory examination of a focused set of putative moderators and mediators of treatment response, including demographics, treatment and medical history, diagnostic-level data for depression and co-morbid conditions, scalar measures of internalizing and externalizing symptoms, family functioning and environment, peer relations, academic and school performance, quality of life, and treatment-related expectancies and beliefs:

#### Clinical Global Impression (CGI)

Blinded assessment of the global severity of the symptoms (CGI-S) at baseline and global change in degree of illness compared to the baseline dichotomized at much improved or better on the Clinical Global Impressions-Improvement (CGI-I) scale [34] rated by an experienced blinded clinical investigator.

#### Children’s Depression Rating Scale-Revised (CDRS-R)

Clinician-rated reduction of depressive symptoms, assessed by a blinded investigator after four weeks of treatment (at T2). The CDRS-R ([35, 36]) is a widely used semi-structured interview to assess clinician-rated depressive symptoms. It has been applied in various multicenter studies to assess efficiency of pharmacotherapy and psychotherapy in adolescents (e.g. TADS, [37]; ADAPT, [38]). It shows good psychometric properties (Cronbach’s Alpha = 0.85; inter-rater reliability = 0.92).

#### Antidepressant medication status

Changes in the treatment regime (initiation/cessation/dose-adjustment of antidepressants) will be assessed weekly from baseline to T2 and in the follow-ups.

#### Sleep quality and effectiveness

Change in sleep quality is assessed via the self-report Sleep-Questionnaire B (SFB/R; [39]), a well validated measure sensitive to treatment changes.

#### Level of activity

Assessed in an objective, ecological valid way via actigraphy (a watch measuring movement and light exposure before/after treatment, assessing time in bed and activity); ecological validity has been proven empirically (e.g. [40]). State-of-the-art software and algorithms will be applied to derive the parameters of interest (e.g. changes in the total degree of activity, time in bed, and sleep efficiency).

#### Health-related quality of life (HRQoL)

Changes in an individual’s perception of their wellbeing are assessed with the KIDSCREEN-10, a short, valid, and reliable self-report, providing a total score reflection of HRQoL [41].

#### Physical activity

The Child Health and Illness Profile-Adolescent Edition (CHIP-AE) is a generic measure of satisfaction with health providing several domains and subdomains. The CHIP-AE is sensitive to treatment effects in adolescents [42]. Here, the domain Resilience with the subdomain Physical Activity shall be assessed, providing information about involvement in a variety of activities related to fitness [43].

#### General psychopathology

Changes in general psychopathology are assessed via self and parent versions of the Strength and Difficulties Questionnaire (SDQ). Its change sensitivity allows estimating treatment efficacy on affective/behavioral symptoms. The validated German version [44] has, for example, been used in the KiGGS survey.

#### Alertness

BLT has direct effects on alertness. Changes in level of alertness will be assessed via the subtest “Alertness” of the Testbattery for Attentional Performance (TAP) [45].

#### Neurobiological indices of circadian function

Two “gold standard” indices of circadian function will be assessed: the DLMOand the cortisol awakening response (CAR). Changes in DLMO from before to after treatment will indicate direct impact of BLT on circadian functioning, while a low pre-treatment CAR has been reported to predict the response to BLT in adults with non-seasonal depression [46].

#### IQ

The German “Zahlen-Verbindungs-Test” (ZVT) [47] is used to assess the intelligence quotient. The ZVT is a trail-making and speed test in which participants are instructed to connect numbers from 1 to 90 which are randomly positioned on a piece of paper. It shows sufficient psychometric properties (test reliability = 0.81–0.97) [48].

#### Seasonal pattern

To control for seasonal effects, the Seasonal Pattern Assessment Questionnaire (SPAQ) will be administered to calculate a total “seasonality score” [49].

### Data analysis

#### Proposed sample size/power calculations

In our pilot study [20], a similar trial design was used, investigating the effects of BLT versus placebo light therapy after a treatment period of 14 days and a follow-up period of three weeks. Remission rates at the end of follow-up (week 5) were 46.7% for BLT and 25.9% for dim light. These remission rates were transformed (2*arcsin; √remission rate). The difference between the transformed remission rates resulted in Cohens h of 0.437 and was used for the calculation of the study sample. A significant difference between the two groups is defined as 3 points on the BDI-II by the NICE guidelines. A standard deviation of 10 points in the BDI-II is assumed. Thus, the proposed study aims to show a group difference of 4.37 points on the BDI-II. Calculations based on this difference yield a sample size of 224 patients (112 for each group) when using an independent t-test at a significance level of 5% and a power of 90%. Sample size was calculated using PROC POWER in SAS® Version 9.4. Drop-outs will be analyzed using multiple imputation methods.

#### Statistical analyses

The primary analysis population is the intention-to-treat (ITT) population comprising all randomized patients. Group difference in change of BDI-II rating under trial treatment will be analyzed by an analysis of covariance (ANCOVA) comprising the covariates baseline BDI-II rating, treatment group, prior pharmacological antidepressant therapy (yes/no), and sex. Missing values will be replaced by multiple imputation. The two-sided level of significance will be α = 5%. Supportive analyses are planned for the Per Protocol Population and the Completer Population. The Per Protocol Population comprises all patients from the ITT population without major protocol violations: lack of compliance with regard to therapy sessions; not meeting all inclusion criteria; meeting at least one exclusion criterion; intake of unallowed concomitant medication during the study; end of treatment visit out of schedule. In an additional sensitivity analysis, missing values will be analyzed based on the last available BDI-II rating (Last Observation Carried Forward [LOCF]). In case the LOCF approach turns out to be not conservative, an additional Baseline Observation Carried Forward (BOCF) analysis will be performed.

Further subgroup analyses are planned to detect possible differences between predictive factors (e.g. trial site, concomitant psychotropic medication for co-morbid disorders, baseline CAR and DLMO, type of depression [seasonal/non-seasonal], season, expectancy questionnaire). No interim analyses are planned. Secondary endpoints will be analyzed descriptively applying appropriate methods depending on the scale of the respective parameter. Interpretation of statistical tests must be exploratory except for the primary outcome.

AEs will be coded according to MedDRA terminology. Detailed information collected for each AE will include: a description of the event; duration; whether the AE was serious; intensity; relationship to trial treatment; action taken; and clinical outcome. Summary tables will present the number of participants observed with AEs by MedDRA System Organ Class and Preferred Term and corresponding percentages. Additional subcategories will be based on event intensity and relationship to trial treatment. A subject listing of all AEs will be prepared.

## Discussion

In this study protocol, we presented the background, design, measurements, and statistical procedures of our randomized, controlled, double-blind multicenter parallel group trial of BLT in a naturalistic inpatient setting. We discussed the medical problems regarding treatment and remission rates in adolescents meeting the criteria of depression and constituted the need to close the gap of evidence of BLT in youth therapy by prolonged (four weeks) intervention time and minimized bias problems due to our randomized, double-blind design.

There are several limitations the study will have to deal with, e.g. the exclusion of the weekends during intervention periods or lack of evidence for actual wearing time of the glasses because of the naturalistic setting. An additional disadvantage is the already regulated sleep-wake rhythm due to the inpatient setting, making a larger difference between activity- and sleep-times among the patients of the two treatment groups difficult to emerge. Moreover, the strict schedules at the infirmaries limit the power of the results compared to a more realistic, ambulant setting.

As remission rates of state-of-the-art treatments for depressed adolescents such as cognitive behavioral therapy and antidepressants remain unsatisfactory, and due to its easy application, low side effects, and optimized compliance, BLT seems to be a promising add-on therapy to psychological and pharmacological approaches to increase standard treatment effects. Since previous studies investigating the effect of BLT in depressed adolescents suffer from small sample sizes, the results of this projected trial will be of great importance.

### Trial status

Recruitment is planned to begin in February 2018.

## Additional file

Additional file 1: SPIRIT 2013 Checklist: Recommended items to address in a clinical trial protocol and related documents*. (DOCX 54 kb)

## Abbreviations

ANCOVA: Analysis of covariance
BDI-II: Beck Depression Inventory II
BLT: Bright light therapy
BOCF: Baseline Observation Carried Forward
CAR: Cortisol awakening response
CDRS - R: Children’s Depression Rating Scale – Revised
CGI-I: Clinical Global Impression-Improvement
CGI-S: Clinical Global Impression-Severity
CHIP – AE: Child Health and Illness Profile-Adolescent Edition
DALYs: Disability Adjusted Life Years
DGKJP: German Association for Child and Adolescent Psychiatry
DLMO: Dim light melatonin onset
HRQoL: Health-related quality of life
ICH-GCP: International Council for Harmonization of Technical Requirements for Pharmaceuticals for Human Use (ICH) - Good Clinical Practice
ITT: Intention-to-treat
IZKS: Interdisziplinäres Zentrums Klinische Studien
KIGGS: Studie zur Gesundheit von Kindern und Jugendlichen in Deutschland
LOCF: Last Observation Carried Forward
MedDRA: Medical Dictionary for Regulatory Activities
MEQ: Morningness–Eveningness Questionnaire
NICE: National Institute for Health and Care Excellence
PCC: Placebo control condition
SAD: Seasonal affective disorder
SAE: Serious adverse events
SDQ: Strengths and Difficulties Questionnaire
SF-B/R: Schlaffragebogen B – Revidierte Fassung
SPAQ: Seasonal Pattern Assessment Questionnaire
T1: Pre-treatment assessment
T2: Post-treatment assessment after four weeks
T3: Post-treatment assessment after three months
T4: Post-treatment assessment after six months
TADS: Treatment of adolescents with depression study
TAP: Testbatterie zur Aufmerksamkeitsprüfung
TAU: Treatment as usual
TORDIA: Treatment of resistant depression in adolescents
UKE: Universitätsklinikum Hamburg-Eppendorf
